# Feedback after OSCE: A comparison of face to face versus an enhanced written feedback

**DOI:** 10.1186/s12909-021-02585-z

**Published:** 2021-03-24

**Authors:** Chin Fang Ngim, Paul Douglas Fullerton, Vanassa Ratnasingam, Valliammai Jayanthi Thirunavuk Arasoo, Nisha Angela Dominic, Cindy Pei Sze Niap, Sivakumar Thurairajasingam

**Affiliations:** Jeffrey Cheah School of Medicine and Health Sciences, Monash University, Johor Bahru, Malaysia

**Keywords:** Assessment, OSCE, feedback, written, face to face, culture

## Abstract

**Background:**

The Objective Structured Clinical Exam (OSCE) is a useful means of generating meaningful feedback. OSCE feedback may be in various forms (written, face to face and audio or video recordings). Studies on OSCE feedback are uncommon, especially involving Asian medical students.

**Methods:**

We compared two methods of OSCE feedback delivered to fourth year medical students in Malaysia: (i) Face to face (FTF) immediate feedback (semester one) (ii) Individualised enhanced written (EW) feedback containing detailed scores in each domain, examiners’ free text comments and the marking rubric (semester two). Both methods were evaluated by students and staff examiners, and students’ responses were compared against their OSCE performance.

**Results:**

Of the 116 students who sat for both formative OSCEs, 82.8% (n=96) and 86.2% (n=100) responded to the first and second survey respectively. Most students were comfortable to receive feedback (91.3% in FTF, 96% in EW) with EW feedback associated with higher comfort levels (p=0.022). Distress affected a small number with no differences between either method (13.5% in FTF, 10% in EW, p=0.316). Most students perceived both types of feedback improved their performance (89.6% in FTF, 95% in EW); this perception was significantly stronger for EW feedback (p=0.008). Students who preferred EW feedback had lower OSCE scores compared to those preferring FTF feedback (mean scores ± SD: 43.8 ± 5.3 in EW, 47.2 ± 6.5 in FTF, p=0.049). Students ranked the “marking rubric” to be the most valuable aspect of the EW feedback. Tutors felt both methods of feedback were equally beneficial. Few examiners felt they needed training (21.4% in FTF, 15% in EW) but students perceived this need for tutors’ training differently (53.1% in FTF, 46% in EW)

**Conclusion:**

Whilst both methods of OSCE feedback were highly valued, students preferred to receive EW feedback and felt it was more beneficial. Learning cultures of Malaysian students may have influenced this view. Information provided in EW feedback should be tailored accordingly to provide meaningful feedback in OSCE exams.

**Supplementary Information:**

The online version contains supplementary material available at 10.1186/s12909-021-02585-z.

## Background

Feedback is the information provided regarding aspects of one’s performance or understanding by an agent and is considered among the most critical influences on a student’s learning [1]. In an extensive review of general education involving 500 meta-analyses and millions of students, Hattie and Timberley found that over 100 factors might influence achievement with an effect size of 0.40 on average but the effect size for feedback alone was 0.79 which ranked feedback among the top influences with a powerful effect on learning [1, 2].

Medical students may receive feedback in a variety of clinical and non-clinical contexts where opportunities for feedback on clinical competencies are especially valuable. With this regard, an Objective Structured Clinical Examination (OSCE) can offer a unique opportunity to provide feedback on clinical competencies. During an OSCE, students rotate through structured stations designed to assess competencies and examiners assess their performance objectively using predetermined criteria [3]. This offers a valuable opportunity to provide feedback which is personalised and timely to help students improve their clinical skills.

Feedback after OSCE has been provided in various ways. For instance, additional time allocated for face to face feedback from examiners immediately after the OSCE has been reported [4–6]. Audio or video recordings of examiners providing generic feedback or personalised feedback have also been utilised [7–9]. Written OSCE feedback given in the form of individual score sheets and examiner comments were reported in a number of studies as well [7, 9, 10].

These studies on OSCE feedback were conducted in Western countries with limited literature from Asian countries where students’ acceptance to the various forms of feedback may differ due to cultural factors. Asian learners reportedly preferred feedback which is indirect and implicit, more group focussed and less self-level feedback compared to learners from Western countries who are more likely to use direct inquiry to seek feedback and generally preferred more direct feedback with focus on the individual’s effort [1, 10, 11].

We conducted this study on OSCE feedback in Malaysia amongst medical students of Monash University Malaysia studying at the Jeffrey Cheah School of Medicine and Health Sciences. Monash University Malaysia is part of the overseas campus of the Monash University in Australia; established in 1998, it is now one of the largest private universities in Malaysia. The School of Medicine was later established in the Malaysian campus in year 2007. Sharing the same educational philosophies, curriculum and assessments as the main campus in Australia, rigorous efforts are especially taken to ensure the equivalence of assessments across different sites. The majority of our students are Malaysians but up to 15% are international students, coming from other parts of Asia, Africa, Europe and Canada.

The study’s aims were: 1. To compare our medical students’ perceptions of two methods of providing OSCE feedback. 2. Examiners’ perceptions were also sought. 3. To examine if students’ feedback preferences related to their OSCE performance.

## Methods

### Study setting

OSCE is employed as a method of summative assessment at the end of the second, third and fourth years in the medical curriculum in Monash University. The summative OSCE exams at the main campus located in Victoria, Australia and the branch campus in Malaysia are identical and conducted synchronously. In year 4, students were tested in four major disciplines: children’s health, women’s health, psychiatry and general practice. Students are assessed in skills such as history taking, physical examination, clinical reasoning, management and communication skills with roles played by simulated patients. They are given four minutes to read a stem and prepare themselves before entering the OSCE station where they spend eight minutes with the examiner and the simulated patient.

For medical students in the Malaysian campus of Monash University, two additional formative OSCEs were organised every year in addition to the summative OSCE described above. The first formative OSCE is usually conducted at the end of semester 1 (May) with students rotating through the two stations covering both the disciplines they have been taught in the first half of the year. A second formative OSCE is carried out at the end of semester 2 (October) consisting of four stations with one station per discipline.

Feedback for the formative OSCE was provided to our students in two ways prior to this study. Firstly, each student received a summary of their performance in each station which stated their total mark, pass/fail status and the pass mark for each station. Information on the whole cohort performance in terms of range of marks, median mark and the 25^th^ percentile mark for each station was also provided. This written feedback is emailed to students within two weeks of the OSCE. Secondly, a post-OSCE generic briefing in the form of a mini-lecture is provided to the whole cohort by the academic staff from each discipline.

### Study participants

This was an interventional study involving Year 4 Monash University Malaysia medical students based at the Clinical School in Johor Bahru, Malaysia. There were a total of 119 students in this cohort when we conducted the study in 2017. The majority were Malaysians (n=114, 96%) with five international students (three from Sri Lanka, one from Uganda and one from Bangladesh). A total of 116 students sat for both the formative OSCEs in semester one and two.

### Study design and intervention

In addition to the existing feedback approach described, we introduced two methods of OSCE feedback as our intervention.

#### Method 1: Face to face (FTF) feedback

This was employed for the semester one OSCE (May 2017). After students completed the tasks in the station, they spent an additional two minutes in the station while the examiner provided them with face to face (FTF) feedback concerning their performance. This duration of two minutes has been shown to be sufficient without unduly prolonging testing time [4]. In summary, students underwent four minutes of preparation time outside the station, eight minutes examination time in the station followed by an additional two minutes FTF feedback with the examiner.

#### Method 2: Enhanced written (EW) feedback

**Fig. 1:**
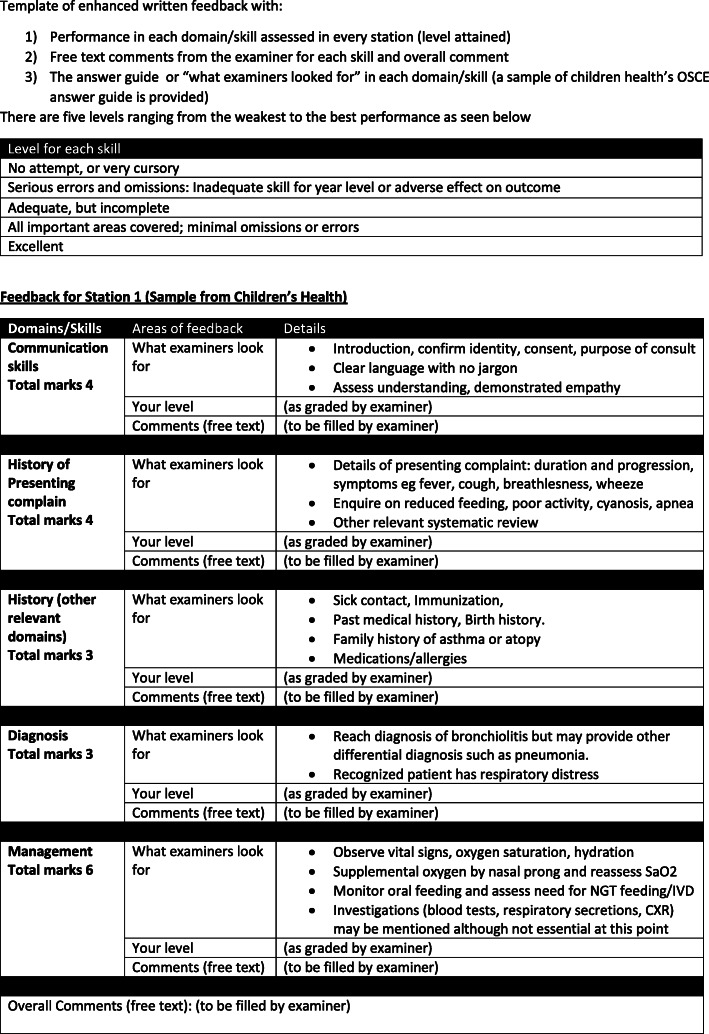
Template of enhanced written feedback

This was employed for the semester two OSCE (October 2017). This enhanced written feedback has three additional areas as shown in Figure 1.

1. A detailed breakdown of their performance in each domain of a station (eg history taking, physical examination, communication skills, diagnosis or management) in terms of grades provided by the examiner on the electronic marking grid.
2. Free text comments: Examiners could type in free text comments under any domain or provide an overall comment during the four minutes students’ preparatory time outside the station. Therefore, each OSCE cycle remained as 12 minutes (four minutes preparation time and eight minutes in the station).
3. The marking rubric to the station with the criteria for each domain under the heading “what examiners look for” (see Figure 1). This information had always been part of the marking rubric provided to examiners only.

Information for the EW feedback was easily retrieved from the existing electronic marking sheet which used Google forms. We collated all the information and used “mail merge” to send individualised feedback to each student.

Our examiners did not receive any formal training on the provision of feedback. They received a set of written instructions in which they were advised to give feedback which were respectful, balanced, actionable and specific with the aim to guide students’ learning and improve their future performance.

### Outcome measures

Students’ and examiners’ perceived quality and utility of the feedback was measured with questionnaires which utilised a five-point Likert scale (1- Strongly Disagree, 2- Disagree, 3- Neutral, 4- Agree, 5-Strongly agree). The survey instruments were adopted from published literature [5, 7, 9] and developed further for this study (available in supplementary folder). All responses were anonymised with the following exception where students were given the option to provide their 7-digit student number in the second survey to allow the lead researcher to match their survey response to their OSCE performance.

The survey instruments were distributed to all students after they received the feedback in both the formative OSCEs in May and October. Survey forms were also disseminated to all examiners after the OSCE. Written consent was obtained from all the students and examiners who participated in this study. Statistical analyses were conducted using IBM® SPSS®Statistics 23.0 in which t-test was used to compare the responses on the Likert scales in FTF versus EW feedback. This study was approved by the Monash University Human Research Ethics Committee (Project no 11168)

## Results

Of the 116 students who sat for both OSCEs, 96 students participated in the survey on face to face (FTF) feedback in semester 1 (response rate 82.8%) and 100 responded to the survey on enhanced written (EW) feedback in semester 2 (response rate 86.2%). As for examiners, there were 14 and 20 respondents respectively.

Survey responses are depicted as the proportion of students who were in agreement with the statements and students’ mean response based on the 5-point Likert scale. As noted from Table 1, the majority of students agreed that the feedback was useful for improving their future performance (89.6% in FTF and 98% in EW) where stronger agreement was seen for EW feedback when we studied the mean responses of the students (p= 0.008) and proportion of students who agreed(p=0.031). Most students also felt comfortable to receive both forms of feedback with EW feedback scoring higher (p=0.022) in mean responses thus implying a higher level of comfort for the written feedback. Only a small group of students felt distressed on receiving the feedback (13.5% in FTF, 10% in EW) with no significant differences between both methods (p=0.316). About three-quarters of students felt the examiners were well prepared to deliver the feedback but interestingly about half of them perceived that examiners should receive more training in both methods of feedback.

**Table 1:** Medical students’ evaluation on both methods of OSCE feedback

Survey items	Responses in agreement^a^ n (%)		*p* value	Mean Response		
	Face to face (*n*=96)	Enhanced Written (*n*=100)		Face to face Mean (SD)	Enhanced Written Mean (SD)	*p* value
The tutors were well-prepared to provide the feedback	72 (75.0)	82 (82)	0.308	3.86 (0.958)	4.04 (0.695)	0.146
The tutors need training to provide this feedback effectively	51 (53.1)	46 (46)	0.315	3.59 (1.010)	3.33 (0.975)	0.075
I felt comfortable about receiving this feedback	88 (91.3)	96 (96)	0.334	4.36 (0.835)	4.61 (0.634)	0.022
I felt distressed after receiving this feedback	13 (13.5)	10 (10)	0.584	2.26 (1.136)	2.10 (1.096)	0.316
I was provided balanced feedback (including both positive and less positive aspects)	71 (74.0)	78 (78)	0.620	3.77 (1.100)	3.97 (0.870)	0.163
In general, the benefits of providing this feedback outweighs the disadvantages	86 (89.6)	95 (95)	0.247	4.44 (0.751)	4.63 (0.580)	0.047
Overall, this feedback was useful to improve my future OSCE performances	86 (89.6)	98 (98)	0.031	4.42 (0.777)	4.67 (0.518)	0.008

Responses were on a 5-point scale: 5-strongly agree, 4-agree, 3- neutral, 2-disagree and 1-strongly disagree. ^a^ Responses of “strongly agree” and “agree” were grouped as “Responses in Agreement”.

We asked the 100 students participating in the second survey on their preference for either method of feedback. 75 (75%) preferred the EW feedback whereas 23 (23 %) students preferred FTF feedback (there were two non-responders). There were 68 students who volunteered their student number to allow the lead researcher to link their responses to their marks in the second formative OSCE. Out of a total score of 80, students who preferred EW feedback had significantly lower scores (mean ± SD: 43.8 ± 5.3, n= 55) compared to those who preferred FTF feedback (mean ± SD: 47.2 ± 6.5, n= 13, p=0.049)

The students were asked to rank which aspects of the enhanced written feedback they found the most valuable to the least valuable. Analysis using Friedman test showed that the most highly ranked was the marking rubric or “what examiners look for” (mean rank 1.66) followed by examiner’s free text comments (mean rank 2.12) and their breakdown of scores in the marking rubric (mean rank 2.21). There was an overall statistically significant difference between the mean ranks ( χ^2^(2) = 17.082, *p* <0.001). Post hoc analysis with Bonferroni correction showed a significant difference in ranking between “marking rubric” and “free text comments” (*Z* =-3.031 , *p* = 0.002) and between “marking rubric” and “breakdown of scores” (*Z* =-3.854 , *p* <0.001). However, there was no difference between “free text comments” and “breakdown of scores” (*Z* = -0.483, *p* = 0.629). For FTF feedback, the duration of two minutes was found to be sufficient for 68 students (71%) whilst the remainder found the duration to be inadequate. The majority of students (91%) found the information that we put together in the EW feedback (grades in each domain, free text comments and marking rubric) to be adequate.

Our examiners were academic staff involved in teaching the students. The examiners’ evaluation on both methods feedback is summarised in Table 2.

**Table 2:** Examiners’ evaluation on both methods of OSCE feedback

Survey Items	Responses in agreement. n (%)			Mean responses		
	Face to face (n= 14)	Enhanced Written (*n*=20)	*p* value	Face to face Mean (SD)	Enhanced Written Mean (SD)	*p* value
I felt well-prepared to provide the feedback	13 (92.9)	14 (70.0)	0.234	4.07 (0.475)	3.75 (0.851)	0.170
I need training to provide this feedback effectively	3 (21.4)	3 (15.0)	0.900	2.77 (1.013)	2.65 (0.875)	0.722
Students are/will be comfortable about receiving this feedback	9 (64.3)	16 (80.0)	0.772	3.92 (0.954)	3.85 (0.671)	0.797
Students are/will be distressed after receiving the feedback	3 (21.4)	0 (0)	0.133	2.50 (1.092)	2.21 (0.535)	0.373
I provided balanced feedback (including both positive and less positive aspects)	12 (85.7)	13 (65)	0.341	4.07 (0.616)	3.65 (0.671)	0.072
In general, the benefits of providing this feedback outweighs the disadvantages	9 (64.3)	17 (85.0)	0.518	3.85 (1.281)	4.05 (0.605)	0.542
The feedback appeared useful to improve students’ future OSCE performances	10 (71.4)	17 (85.0)	1.000	4.08 (1.165)	4.15 (0.671)	0.838
I had adequate time to provide this feedback	10 (71.4)	15 (75.0)	0.931	3.79 (1.122)	3.84 (0.834)	0.869

Responses were on a 5-point scale: 5-strongly agree, 4-agree, 3- neutral, 2-disagree and 1-strongly disagree. Responses of strongly agree and agree were grouped as “Responses in Agreement”

In general, examiners felt prepared in providing feedback (92.9% in FTF, 70% in EW) and only a small proportion perceived the need for further training in providing feedback (21.4% in FTF, 15% in EW). 85% of tutors felt the EW feedback was beneficial in terms of improving performance compared to 71.4% for FTF feedback. About three quarters of examiners felt they had enough time to complete the feedback (71.4% in FTF, 75% in EW). In general, there were no significant differences between both types of feedback from the examiners’ point of view. Of the 14 examiners who had delivered both types of feedback, eight preferred the EW format and six preferred FTF feedback.

## Discussion

The role of assessment to determine if students have achieved certain knowledge and skill sets (assessment *of* learning) has shifted to “assessment *for* learning” in recent decades. This was proposed as early as 1989 [12] . Assessment *for* learning is an approach in which the assessment process is maximally information-rich and embedded in the educational process to steer and foster learning of each individual student [13]. Effective feedback is therefore an integral aspect of this assessment approach.

Feedback refers to information describing the students’ performance in a given activity meant for guiding their future performance and hence is a key step in the acquisition of clinical skills [14]. From our study, OSCE feedback in the form of either FTF or an EW format was generally perceived to be beneficial and both methods were well-received by both students and examiners. In comparing both methods of feedback, there was a perception that EW feedback was more beneficial in improving future performance and students felt more comfortable in receiving feedback in the written form over FTF feedback.

The utility of written feedback for OSCE has been described in several studies. In one of the first publications on OSCE feedback, students were given their marked checklists immediately after the OSCE and this was followed by either observing another student’s performance or watching a video tape of an experienced examiner [7]. Taylor and Green compared two methods of written feedback; the first was skills-based where students’ performance in all the OSCE stations were collapsed into seven skills areas (eg history taking, examination) and graded accordingly [15]. The second intervention (station based) consisting of examiners’ comments on students’ performance on each task was rated higher in terms of students’ satisfaction although subsequent performances did not differ between both groups. The authors concluded that skills-based feedback may not be specific enough for students to improve upon whereas examiners’ comments may have been too evaluative of the student rather than the task itself [15].

Students appeared to value detailed information about the individual OSCE stations and this was reflected in our study where the EW feedback was preferred. The marking rubric was regarded as the most valuable item in the written feedback as it provided the expected answers thus enabling students to identify their gaps with ease. Marking rubrics may be disseminated in a formative OSCE as a means to aid students’ learning but this practice may be more restricted in a high-stakes, summative OSCE due to test security restrictions and institutional policies. The release of the criteria in the marking rubrics or the “expected answers” can be both beneficial and problematic. On one hand, it may encourage deep learning if the students attempt to fully understand its context and conversely, its provision may encourage other students to memorise a collection of isolated facts without engaging an active learning process.

Our students found that examiners’ free text comments were quite useful and this form of feedback was ranked second after “provision of marking rubric”. As feedback should be specific and document the student’s strengths and areas for improvement, the comments by our examiners may be lacking as 44% of students perceived a need for examiners’ training in this respect. It is also possible that some examiners faced challenges putting their thoughts in written form within the time frame as about a quarter of examiners felt there were inadequate time. In this respect, the use of audio feedback in which voice recordings of the examiners are delivered to students after the OSCE [8] enabling students to receive personalised and detailed feedback is a viable alternative option.

The rich information from the scoring matrix used for the OSCE can be maximised as a form of feedback. Harrison described an interactive website used to deliver summative OSCE feedback in various forms to the students: either station by station or on domains common across stations with information on the students’ performance and graphical comparison with the entire cohort [16]. Whether this form of information-rich feedback will improve performance should be evaluated as compared to feedback through comments, provision of marks or grades alone may have little influence on future performance [17, 18]. Our students rated the provision of information on their grades in the scoring matrix as the least useful type of written feedback and this is probably due to lack of specific details for them to identify their gaps. In fact, the provision of marks in addition to comments has been reported to reduce the value placed on the comments provided by examiners [1, 18].

Face to face feedback has been used in OSCE [4–6]. Time constraints may be prohibitive and, in our study, the inclusion of two minutes to each 12 minutes cycle in an OSCE prolonged testing time by 16%. In the study by Hodder, 2 minutes of immediate feedback significantly improved competency in the performance of criterionbased task, at least over the short term where retesting occurred immediately after the feedback [4]. However, the challenges of retaining and integrating information from FTF feedback may be compounded by factors such as the anxiety it may provoke and distraction as students need to move to another station with new tasks. Poor recall of the content of the immediate verbal feedback items has also been reported. In a study where residents were given two minutes of verbal feedback during their OSCE, they recalled very few feedback points immediately after the OSCE and one month later and what they recalled were not reflective of the actual feedback given [6]. Poor recall of verbal feedback received during the OSCE was also reported among dental residents when retested two months later [19].

This does not necessarily imply that it is of limited value. In fact, immediate face to face feedback is still highly valued especially by “Western” learners due to the opportunity to have an interactive exchange with examiners and gaining a deeper understanding of an individual’s performance [11]. As the majority of our students were Malaysians (96%), cultural influences may have contributed to the preference for written feedback over face to face feedback. Studies have shown that Asian learners prefer teacher-centred learning where teachers provide the necessary information and students’ communication with teachers is implicit and indirect whereas learners from Western countries prefer student-centred learning and they value the opportunity for explicit verbal communication and independent learning [10, 20]. The EW feedback we provided fits the Asian students’ learning approaches hence contributing to students’ perceptions on its benefits.

Interestingly, the minority group of students in our study who preferred face to face feedback performed better in the OSCE exam. There are several potential explanations for this observation.

- Self-confident, strong performers did not receive challenging FTF feedback
- The OSCE examination format matched the active, student-centred learning approaches of those who preferred FTF feedback.
- In general, Asian students are “face conscious” and fear embarrassment especially when their knowledge gaps are exposed [21] hence poor performers preferred to avoid a FTF discussion.

Analysis of the survey results amongst our staff examiners showed no significant difference on their views of either method of feedback although inferences are limited by the small number of respondents. It is interesting to note however, most examiners (92.9%) felt prepared to provide FTF feedback in comparison to students’ perception on examiners’ preparedness (75%). Similarly, only a small percentage of examiners (21.4% in FTF, 14% in EW) felt that they required further training to provide feedback in contrast to a larger percentage of students who perceived that examiners required training (53.1% in FTF, 44% in EW). Although we did not audit the quality of feedback, the incongruence between students’ and examiners’ perceptions suggests that formal training for examiners may be beneficial or that student feedback literacy needs development.

All in all, both feedback methods are highly valued. Due to time constraints and ease of dissemination, the EW feedback is now an embedded feature in our school’s formative OSCEs. We feel that it meets the requirements of effective feedback that influences learning such as knowledge of appropriate standards by both learners and teachers, comparison of one’s own work with these standards and enabling actions to close the gap between the two [22]. Explicit assessment criteria can directly pave the way for self-regulation in learning to occur where the transparency provided by rubrics can clarify students’ expectation and allow them to experience greater perceived control and confidence [23]. It is important however, to ensure that students get the opportunity to clarify misconceptions as feedback is more powerful when it addresses faulty interpretation leading to the development of efficient strategies for processing and understanding the material [1]. In addition to using effective feedback approaches, the development of students' assessment literacies is also vital to address differential attainment and improve students' learning [24]

Our study has several limitations. We only measured students’ and examiners’ perceptions towards both methods of feedback which may not translate into actual future performance. Reviewing their performance in the summative OSCE may gauge the impact of feedback but would not have distinguished which method worked better as students received both forms of feedback. FTF feedback was based on two OSCE stations, while EW feedback followed four OSCE stations. Potentially this may have influenced student views on the feedback. The preference for EW feedback in our study was likely due to the large amount and relevance of information students received encompassing three areas as compared to the verbal FTF feedback from examiners. Direct comparison of examiners’ feedback (verbal versus written) was not addressed. This study was conducted at a single site which is a Malaysian campus of an Australian university which may limit its generalizability. That said, our findings contribute to the limited data on OSCE feedback from an Asian country. The data may be useful in informing local institutions as well as those from Western countries which are striving to be more inclusive in their learning and assessment approachesTo explore the students’ perception further, focus group discussions could be useful. Lastly, it is important to study how students utilise the feedback provided to them. Although feedback is the most powerful single moderator that enhances achievement, the self-strategies that students develop can significantly alter the consequences of this feedback [25].

## Conclusion

The OSCE can be useful as a tool for both assessment-for-learning and assessment-of-learning where the opportunity to generate meaningful feedback to improve clinical competence has long been recognised. Medical students highly value feedback from OSCE exams in any form and consistently request additional feedback. In our study which compared face to face feedback and enhanced written feedback, our medical students showed a stronger preference for the written format. This observation may be explained by the richness of the information we provided in our EW feedback as well as influences from our Asian learners’ cultural background.

Without the limitation of time, financial considerations, logistic and test security issues, OSCE feedback should be delivered in a variety of methods to consolidate the various learning approaches adopted by students. Where those limitations exist, medical schools should evolve to utilise available technologies to deliver feedback that is meaningful in a prompt manner. In this context, an enhanced written feedback may be the easiest and most cost-effective as it utilises information which is readily available and does not increase testing time. Students’ acceptance and the influence of feedback on their future performance from various regions merits more studies and will be useful to inform assessment policies and future research.

## Supplementary Information

**Additional file 1.**

## Data Availability

The data that support the findings of this study are available from the corresponding author via E-mail upon reasonable request and permission

## Abbreviations

OSCE: Objective Structured Clinical Exam
FTF: Face to face
EW: Enhanced written
