# Supplementary material for: Feedback after OSCE: A comparison of face to face versus an enhanced written feedback
Source: BMC Med Educ. 2021 Mar 24;21:180. doi: 10.1186/s12909-021-02585-z (PMC7992790; doi:10.1186/s12909-021-02585-z)
Supplement: Supplementary file 1 — Additional file 1. [file 12909_2021_2585_MOESM1_ESM.docx]

**Supplementary Folder**

**Survey on OSCE feedback (For Students)**

Please put a tick (√) at the column ranging from Strongly Disagree to Strongly Agree

|  | Strongly Disagree | Disagree | Neutral | Agree | Strongly Agree |
| --- | --- | --- | --- | --- | --- |
| The tutors were well-prepared to provide the feedback |  |  |  |  |  |
| The tutors need training to provide this feedback effectively |  |  |  |  |  |
| I felt comfortable about receiving this feedback |  |  |  |  |  |
| I felt distressed after receiving this feedback |  |  |  |  |  |
| I was provided balanced feedback (include both positive and less positive aspects) |  |  |  |  |  |
| In general, the benefits of providing this feedback outweighs the disadvantages |  |  |  |  |  |
| Overall, this feedback was useful to improve my future OSCE performances |  |  |  |  |  |

Additional questions for

**Survey in Semester 1 (Face to Face feedback):**

1. *The duration of 2 minutes to receive face to face feedback is: □ Too short □ Just adequate□ Too long*

**Survey in Semester 2 (Enhanced written feedback)**

1. *The information you received from this written feedback was □ Adequate □ Inadequate*
2. *You have now undergone two methods of OSCE feedback in Semester 1 and Semester 2. Which do you prefer?*

*□ Face to face feedback □ Enhanced written feedback*

1. *Please rank from 1 to 3 which aspects of the enhanced written feedback you found the most valuable to the least valuable (1 for “most valuable” to 3 for “least valuable”)*

*□ breakdown of your scores in the individual domains of every station.*

*□ examiner’s free text comments*

*□ answer guide or “what examiners look for”*

1. *Student ID (Optional)....................Provision of your ID will allow the lead researcher to link your response to your OSCE marks.*

**Survey on OSCE feedback (For Examiners)**

Please put a tick (√) at the column ranging from Strongly Disagree to Strongly Agree

|  | Strongly Disagree | Disagree | Neutral | Agree | Strongly Agree |
| --- | --- | --- | --- | --- | --- |
| I felt well-prepared to provide the feedback |  |  |  |  |  |
| I need training to provide this feedback effectively |  |  |  |  |  |
| Students are/will be comfortable about receiving this feedback |  |  |  |  |  |
| Students are/will be distressed after receiving the feedback |  |  |  |  |  |
| I provided balanced feedback (including both positive and less positive aspects) |  |  |  |  |  |
| In general, the benefits of providing this feedback outweighs the disadvantages |  |  |  |  |  |
| The feedback appeared useful to improve students’ future OSCE performances |  |  |  |  |  |
| I had adequate time to provide this feedback |  |  |  |  |  |

Additional questions for

**Survey in Semester 2**

1. *For examiners who had the opportunity to provide two methods of OSCE feedback in Semester 1 (face to face) and Semester 2 (enhanced written feedback), which do you prefer?*

*□ Face to face feedback □ Enhanced written feedback*
